# *In vivo* visualization of fluorescence reflecting CDK4 activity in a breast cancer mouse model

**DOI:** 10.1002/mco2.136

**Published:** 2022-06-10

**Authors:** Yi‐Yang Gao, Rui‐Qin Yang, Kang‐Liang Lou, Yong‐Ying Dang, Yuan‐Yuan Dong, Yue‐Yang He, Wen‐He Huang, Min Chen, Guo‐Jun Zhang

**Affiliations:** ^1^ Department of Breast and Thyroid Surgery Xiang'an Hospital of Xiamen University, School of Medicine, Xiamen University Xiamen China; ^2^ Fujian Key Laboratory of Precision Diagnosis and Treatment in Breast Cancer Xiang'an Hospital of Xiamen University, Xiamen University Xiamen China; ^3^ Xiamen Key Laboratory of Endocrine‐Related Cancer Precision Medicine Xiang'an Hospital of Xiamen University, Xiamen University Xiamen China; ^4^ Xiamen Research Center of Clinical Medicine in Breast and Thyroid Cancers Xiang'an Hospital of Xiamen University, Xiamen University Xiamen China; ^5^ Central Laboratory Xiang'an Hospital of Xiamen University, School of Medicine, Xiamen University Xiamen China; ^6^ Cancer Research Center of Xiamen University School of Medicine, Xiamen University Xiamen China

**Keywords:** breast cancer, CDK4 inhibitors, fluorescence imaging, kinase activity, therapeutic evaluation

## Abstract

The CDK4/6‐Rb axis is a crucial target of cancer therapy and several selective inhibitors of it have been approved for clinical application. However, current therapeutic efficacy evaluation mostly relies on anatomical imaging, which cannot directly reflect changes in drug targets, leading to a delay in the selection of optimal treatment. In this study, we constructed a novel fluorescent probe, CPP30‐Lipo/CDKACT4, for real‐time monitoring of CDK4 activity and the therapeutic efficacy of its inhibitor in HR^+^/HER2^–^ breast cancer. CPP30‐Lipo/CDKACT4 exhibited good optical stability and targetability. The signal of the probe in living cells decreased after CDK4 knockdown or palbociclib treatment. Moreover, the fluorescence intensity of the tumors after 7 days of palbociclib treatment was significantly lower than that before treatment, while no significant change in tumor diameter was observed under magnetic resonance imaging. Overall, we developed an innovative fluorescent probe that can monitor CDK4 activity and the early therapeutic response to CDK4 inhibitors in living cells and *in vivo*. It may provide a new strategy for evaluating antitumor therapeutic efficacy in a clinical context and for drug development.

## Graphical abstract

In this study, we utilized a unique targeting delivery strategy that encapsulated the peptide biosensor CDKACT4 into CPP30‐modified DOPE cationic liposomes to obtain a novel and stable fluorescent probe, CPP30‐Lipo/CDKACT4. It realized the visualization of CDK4 activity in living cells and *in vivo*, and initially assessed the therapeutic efficacy of CDK4 inhibitors in the treatment of HR^+^/HER2^‐^ breast cancer in pre‐clinical experiments. This study provides a novel thought for the assessment of clinical drug therapeutic efficacy and accurate guidance of individualized antitumor therapy, as well as offering a new tool for the development of antitumor drugs.

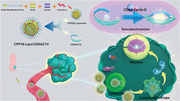

## 1 INTRODUCTION

Dysregulation of the cell cycle is one of the most important hallmarks of cancer.^1^ Cell cycle progression is driven by the phasic expression of cyclins and activation of cyclin‐dependent kinases (CDKs).^2^ Given that the CDK4/6 ‐ retinoblastoma protein (Rb) axis is essential for the transition from G1 to S phase in the cell cycle,^3^ it is a popular drug target in several types of cancer.^4^, ^5^ Multiple clinical trials have shown that selective CDK4/6 inhibitors, such as palbociclib, abemaciclib, and ribociclib, in combination with aromatase inhibitors or fulvestrant can significantly prolong progression‐free and overall survival in hormone receptor (HR)‐positive and human epidermal growth factor receptor 2 (HER2)‐negative metastatic breast cancer patients, and thus have been approved by the FDA and EMA.^5^, ^6^, ^7^, ^8^ Although CDK4/6 inhibitors can effectively improve the prognosis of HR^+^/HER2^–^ breast cancer, there are differences in the sensitivity to CDK4/6 inhibitors among individuals. Some patients show de novo resistance, while others acquire resistance to CDK4/6 inhibitors after a few cycles of therapy. There is thus an urgent need for a precise method to evaluate the therapeutic response to CDK4/6 inhibitors and to differentiate who is likely to be sensitive to those drugs.

To evaluate the therapeutic response to anti‐tumor drugs including kinase‐targeting agents, Response Evaluation Criteria in Solid Tumors1.1 (RECIST1.1)^9^ is the most widely used tool. These criteria are largely based on anatomical imaging modalities, such as computed tomography and magnetic resonance imaging (MRI), or direct measurement of the tumor by a caliper. However, a change in tumor size usually appears after at least two cycles of chemotherapy, leading to a delay in the selection of optimal treatment. It is necessary to evaluate therapeutic response as early as possible so that patients do not receive unnecessary and toxic chemotherapy/targeted therapy. Recently, molecular imaging technology has attracted substantial attention for monitoring the response to treatment with anti‐tumor drugs. In particular, fluorescence molecular imaging allows the repetitive, noninvasive assessment of molecular targets.

CDK4/6 is a serine/threonine kinase, its activity cannot be directly monitored by labeling this protein itself with a fluorophore. Thus, it is crucial to design a special probe that exhibits fluorescence changes upon Rb phosphorylation without the need for separating the substrates and products for analysis.^10^, ^11^ Interestingly, a dye‐conjugated peptide‐based biosensor (CDKACT4) was reported to monitor CDK4 kinase activity through sensitive changes in the fluorescence intensity of cell extracts.^12^ This recombinant peptide biosensor consists of a CDK4‐specific substrate sequence labeled with a solvatochromic fluorophore (5‐TAMRA) and a phosphor‐amino‐acid binding domain (PAABD). PAABD recognizes the phosphorylated amino acid as the substrate phosphorylated by CDK4‐cyclin D, thereby inducing a conformational change of the peptide that alters the environment of 5‐TAMRA, ultimately triggering the enhancement of fluorescence emission. Unfortunately, this biosensor has not been further applied in living cells and *in vivo*. To the best of our knowledge, no studies have yet been conducted to monitor the activity of CDK4 kinase in real‐time using exogenous contrast agents.

Liposomes have long been regarded as ideal and safe drug delivery systems with the ability to be loaded with different types of cargo such as hydrophilic and hydrophobic drugs, protein‐based drugs, peptides, and nucleic acids.^13^, ^14^, ^15^ Incorporation of special components, such as DOPE, in cationic liposomes can effectively achieve lysosomal escape, to avoid lysosomal degradation and deactivation.^16^ Cell‐penetrating peptides (CPPs) have been demonstrated to optimally and specifically target tumor cells and subsequently enter the nucleus,^17^ of which, CPP30 is a representative breast cancer‐homing CPP.^18^

**FIGURE 1:**
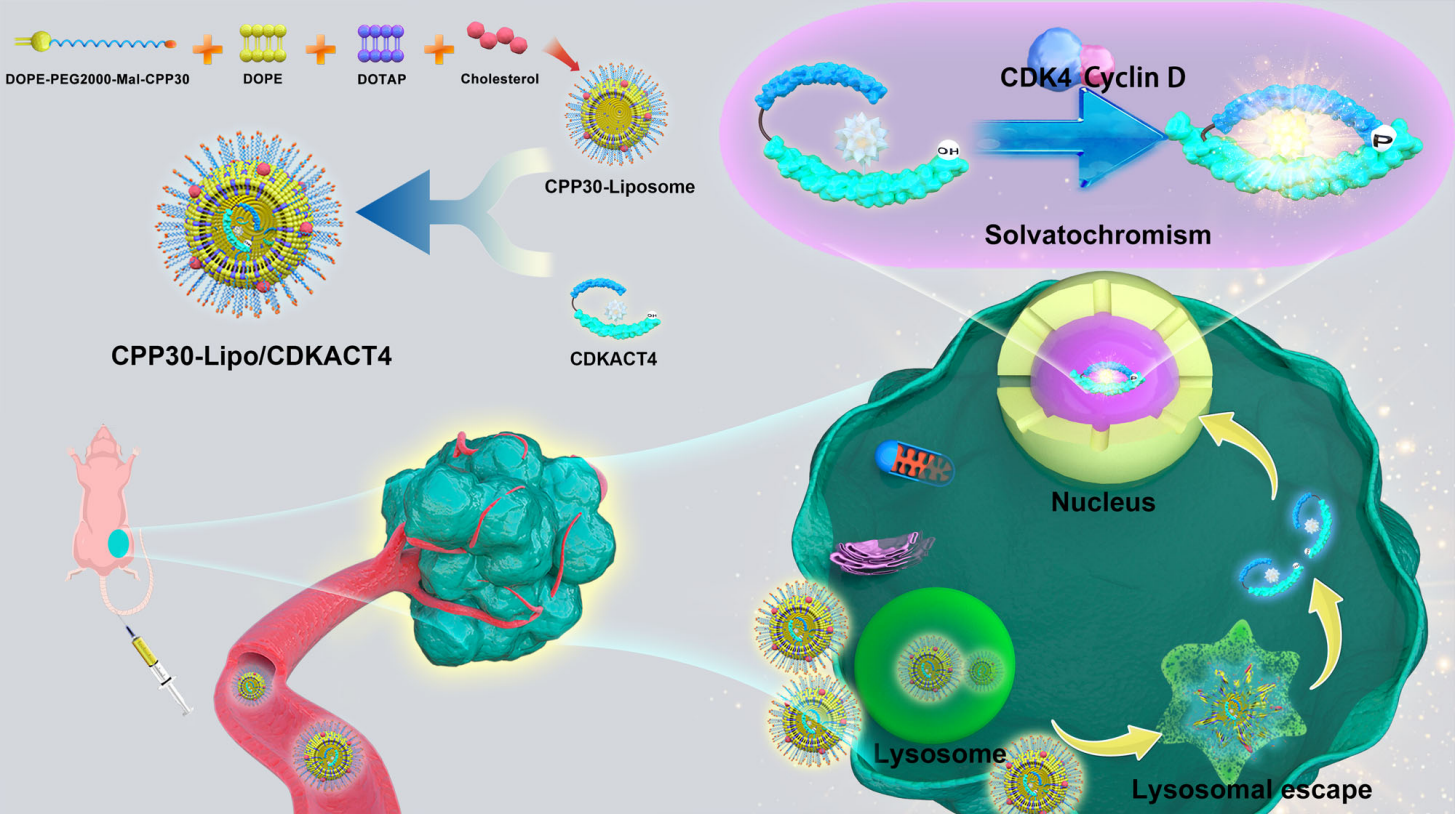
Preparation procedure and workflow of CPP30‐Lipo/CDKACT4 for monitoring CDK4 kinase activity (Created by Adobe Illustrator 2020)

In this study, we took the peptide biosensor CDKACT4 as a core and encapsulated it into CPP30‐modified DOPE cationic liposomes to obtain a novel fluorescent probe, CPP30‐Lipo/CDKACT4. We aimed to achieve the noninvasive monitoring of CDK4 kinase activity and the efficacy of CDK4 inhibitors in living cells and *in vivo* by real‐time fluorescence imaging, acquire an attractive tool for diagnostics, and prediction of pathological progression and response to therapeutics targeting the CDK4 biomarker (Figure 1).

## 2 RESULTS

### 2.1 Synthesis and characterization of CPP30‐Lipo/CDKACT4

**FIGURE 2:**
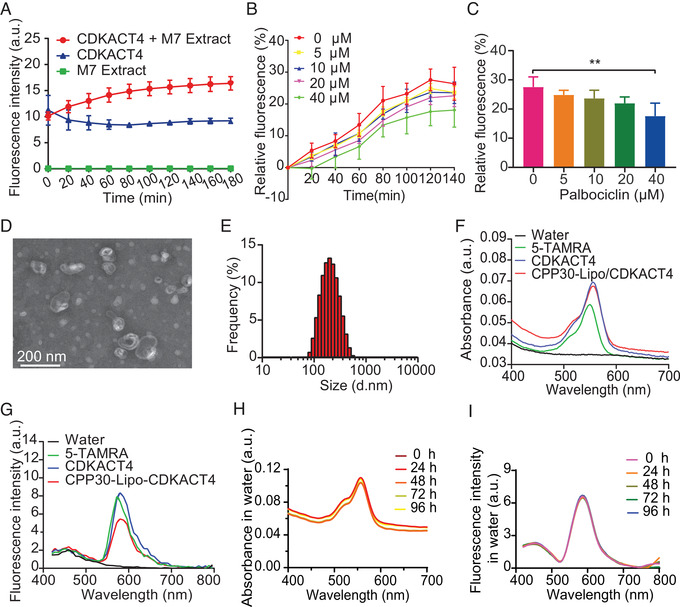
Characterization and spectral stability of CPP30‐Lipo/CDKACT4. (A) Fluorescence changes of CDKACT4 biosensor incubated with MCF‐7 cell extracts (red), CDKACT4 alone (blue), and cell extracts alone (green) during 180 min (*n* = 5). (B) Relative fluorescence intensity of CDKACT4 biosensor incubated with MCF‐7 cell extracts was monitored over time after treatment with different concentrations of palbociclib (*n* = 5). (C) Relative fluorescence intensity of MCF‐7 cell extracts‐incubated CDKACT4 at 120 min post‐treated with different concentrations of palbociclib from (b) (*n* = 5, **p < 0.01). (D) Transmission electron microscopy (TEM) images of CPP30‐Lipo/CDKACT4. Scale bar = 200 nm. (E) Diameter distribution of CPP30‐Lipo/CDKACT4. (F, G) UV–vis absorption spectra (F) and fluorescence emission spectra (G) of water, 5‐TAMRA, CDKACT4, and CPP30‐Lipo/CDKACT4. (H, I) Changes in absorption and fluorescence spectra of CPP30‐Lipo/CDKACT4 during 96 h in water. The samples were stored at room temperature in the dark

We initially determined whether the CDKACT4 peptide biosensor that we synthesized was effective at monitoring CDK4 kinase activity in MCF‐7 breast cancer cell extracts. Before that, HPLC analysis showed that CDKACT4 had high purity of 95.50% (Figure S1D). Then the fluorescence detection showed that this biosensor responded to CDK4/Cyclin D in cell extracts through a robust increase in fluorescence emission (50%–77%) (Figure 2A). Moreover, the fluorescence of the CDKACT4 peptide biosensor decreased in a dose‐dependent manner upon incubation with cells extracts prepared with the CDK4 inhibitor palbociclib (Figure 2B,C).

Next, we synthesized DOPE‐PEG_2000_‐Mal‐CPP30 using DOPE‐PEG_2000_‐Maleimide and C‐CPP30. After purification, the molecular weights of C‐CPP30, DOPE‐PEG_2000_‐Maleimide, and DOPE‐PEG_2000_‐Mal‐CPP30 were determined by matrix‐assisted laser desorption ionization time‐of‐flight mass spectrometry (MALDI‐TOF MS) (Figure S1A–C). This confirmed that the peptide had been successfully linked to phospholipids. Then, the peptide biosensor CDKACT4 was encapsulated into CPP30‐modified liposomes to obtain CPP30‐Lipo/CDKACT4. Transmission electron microscopy showed that CPP30‐Lipo/CDKACT4 was spherical and monodisperse with a diameter between 150 and 200 nm (Figure 2D). The average size of CPP30‐Lipo/CDKACT4 was further characterized as 161 nm by dynamic light scattering (Figure 2E). Besides, the mean polymer dispersity index (PDI) and zeta potential were 0.07 and 60.2, respectively (Table S1).

CDKACT4 and CPP30‐Lipo/CDKACT4 exhibited similar visible absorption and FL emission to 5‐TAMRA (Figure 2F,G). It should be noted that a slight red shift (≈6 nm), relative to 5‐TAMRA, could be observed in CDKACT4 and CPP30‐Lipo/CDKACT4 with a maximum absorption peak at 556 nm, which indicated that 5‐TAMRA had been successfully linked with the lysine residue of the substrate sequence. To clarify optical stability, the absorption and fluorescence spectra of CPP30‐Lipo/CDKACT4 in different solutions (water, phosphate‐buffered saline [PBS], and PBS containing 10% fetal bovine serum [FBS]) were measured. The results showed that CPP30‐Lipo/CDKACT4 maintained spectral stability throughout the 96 h regardless of solutions (Figure 2H,I and Figure S2A–D). Also, in the solvents at different pH values of 5.5, 6.5, and 7.4, the probe CPP30‐Lipo/CDKACT4 possesses good spectral stability (Figure S2E–J). Therefore, different media would not affect the optical characteristics of CPP30‐Lipo/CDKACT4, making it possible to monitor the change in kinase activity more precisely.

Taking these experiments together, it was shown that CPP30‐Lipo/CDKACT4 is an appropriately sized, stable probe for biological application, and has valid components to monitor CDK4 activity in living cells and *in vivo*.

### 2.2 Cellular internalization and cytotoxicity of CPP30‐Lipo/CDKACT4

**FIGURE 3:**
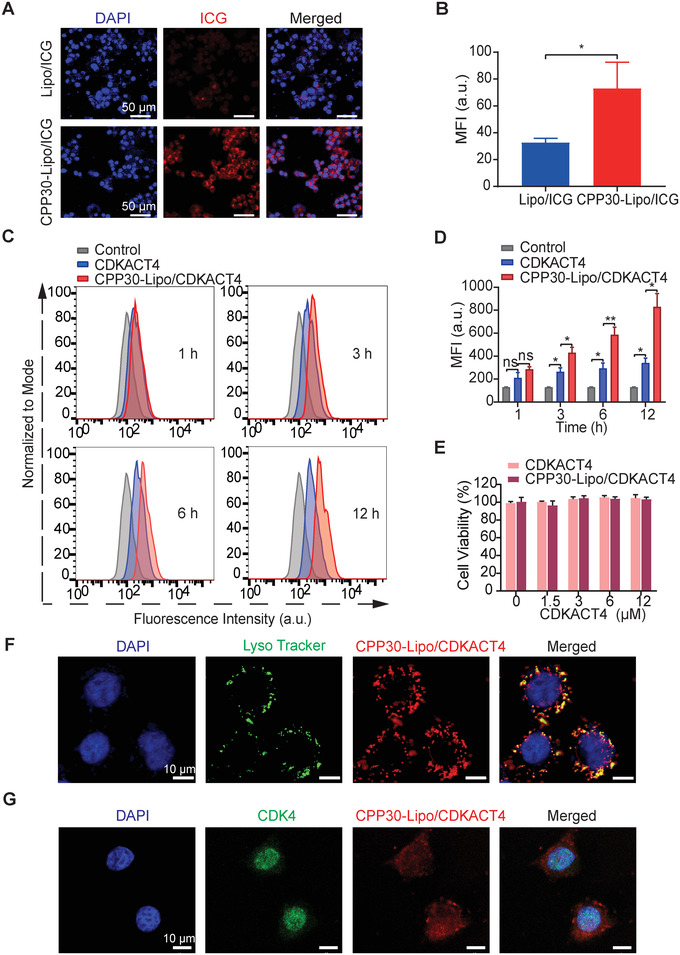
*In‐vitro* intracellular uptake and endosomal escape ability of CPP30‐Lipo/CDKACT4. (A, B) Representative fields under fluorescence microscopy (A) and mean fluorescence intensity (MFI, B) of MCF‐7 cells after incubation with Lipo/ICG or CPP30‐Lipo/ICG. Scale bar = 50 μm. C, Endocytosis of CDKACT4 and CPP30‐Lipo/CDKACT4 by MCF‐7 cells at 1, 3, 6, and 12 h post‐incubation as determined by flow cytometry. (D) Mean fluorescence intensity from flow cytometric analysis (C) at selected time points (*n* = 3, *p < 0.05, **p < 0.01). (E) Viability of MCF‐7 cells after incubation with different concentrations of CDKACT4 and CPP30‐Lipo/CDKACT4 for 72 h (*n *= 5). (F) CPP30‐Lipo/CDKACT4 (red), and Lysotracker‐stained lysosomes (green) were imaged by confocal microscopy at 4 h after CPP30‐Lipo/CDKACT4 and MCF‐7 cell co‐incubation. Nuclei stained by Hoechst 33342 (blue). Scale bar = 10 μm. (G) Subcellular localization of CPP30‐Lipo/CDKACT4 (red) and endogenous CDK4 (green) in MCF‐7 cells as detected by indirect immunofluorescence and imaged by confocal microscopy. Nuclei stained by DAPI (blue). Scale bar = 10 μm

Previous studies demonstrated that CPP30 showed a good ability to target MCF‐7 cells. To explore whether CPP30 retains this ability after linking with lipids, we loaded the CPP30‐Liposome with indocyanine green (ICG). The internalization of ICG into cells was greatly promoted by CPP30 (Figure 3A,B), suggesting that CPP30‐Liposome could markedly facilitate the transport of agents into cells. To compare the homing ability of CPP30‐Lipo/CDKACT4 with that of CDKACT4 in breast cancer cells, MCF‐7 cells were incubated with them for 1, 3, 6, and 12 h. Flow cytometry showed that CPP30‐Lipo/CDKACT4 was taken into MCF‐7 cells more than CDKACT4 (Figure 3C). Quantitatively, the mean fluorescence intensities were significantly higher in CPP30‐Lipo/CDKACT4‐treated cells than in CDKACT4 treated cells from 3 h onwards (*p* < 0.05) (Figure 3D).

A key feature for the peptide biosensor to monitor kinase activity in living cells is the avoidance of endosomal entrapment and digestion. So, we used DOPE cationic liposome as a carrier to deliver CDKACT4. Confocal microscopy analysis showed that only a small proportion of the red signals from CPP30‐Lipo/CDKACT4 overlapped with the green signals from lysosomes, indicating the effective escape of CDKACT4 from the endo‐lysosomes into the cytoplasm (Figure 3F). To explore whether the subcellular localization of CPP30‐Lipo/CDKACT4 overlapped with that of its target, MCF‐7 cells with internalized CPP30‐Lipo/CDKACT4 were further labeled with an antibody against CDK4. Confocal microscopy showed that CPP30‐Lipo/CDKACT4 colocalized with CDK4 in the nucleus (Figure 3G), in line with the need for CPP30‐Lipo/CDKACT4 to monitor the CDK4 kinase activity.

Finally, an MTS assay was performed on MCF‐7 cells to evaluate the cytotoxicity of CPP30‐Lipo/CDKACT4. Cell viability was not impaired by different concentrations of CPP30‐Lipo/CDKACT4, implying that CPP30‐Lipo/CDKACT4 possesses no obvious cytotoxicity (Figure 3E).

Taken together, our results showed that CPP30‐Lipo/CDKACT4 can effectively enter MCF‐7 breast cancer cells, and has good potential for monitoring CDK4 kinase activity in living cells.

### 2.3 CPP30‐Lipo/CDKACT4 reports the cell cycle and CDK4 activity in living cells

**FIGURE 4:**
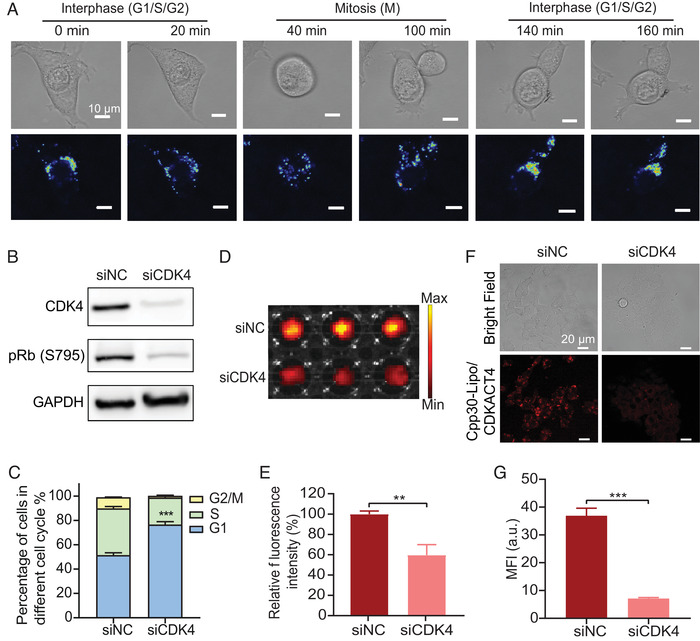
CPP30‐Lipo/CDKACT4 reports the cell cycle and CDK4 activity in living cells. (A) Timelapse micrographs of dividing cells: overlaid images of phase contrast and spectral representation of relative CPP30‐Lipo/CDKACT4 fluorescence intensity. Scale bar = 10 μm. (B) Western blot of CDK4 and pRb (S795) protein levels from MCF‐7 cells treated with siRNA directed against CDK4 (siCDK4) or none (siNC). (C) Cell distribution of siNC‐treated MCF‐7 cells and siCDK4‐treated MCF‐7 cells in different phases of the cell cycle (*n* = 3, ***p < 0.001). (D, E) Fluorescence images (D) and mean fluorescence intensity (MFI) (E) of siNC‐treated MCF‐7 cells and siCDK4‐treated MCF‐7 cells after incubation with CPP30‐Lipo/CDKACT4 (*n* = 3, **p < 0.01). (F, G) Representative fields under fluorescence microscopy (F) and MFI (G) of siNC‐treated MCF‐7 cells and siCDK4‐treated MCF‐7 cells after incubation with CPP30‐Lipo/CDKACT4 (*n* = 3, ***p < 0.001). Scale bar = 20 μm

As a probe containing the substrate sequence derived from Rb as a core, CPP30‐Lipo/CDKACT4 is expected to monitor the cell cycle progression and CDK4 activity. Live‐cell phase contrast and fluorescence time‐lapse microscopic imaging were used to observe the changes in distribution and intensity of CPP30‐Lipo/CDKACT4 fluorescent signals in pace with the changes in cell morphology in different cell cycle phases. As expected, CPP30‐Lipo/CDKACT4 gradually accumulated around the nucleus of MCF‐7 cells during interphase (G_1_/S/G_2_) but dispersed during mitosis (Figure 4A). However, because there are no significant differences in the morphology of cells in the G_1_, S, and G_2_ phases under a microscope, we could not further explore whether the dynamic change of CPP30‐Lipo/CDKACT4 could be seen during the interphase.

To further verify the ability of CPP30‐Lipo/CDKACT4 to quantify the CDK4 activity in living cells, we treated the MCF‐7 cells with siRNA targeting CDK4, leading to a partial reduction of pRb (S795) protein level, as detected by Western blotting (Figure 4B), and a partial G_1_ block in MCF‐7 cells, as assessed by propidium iodide staining followed by FACS (Figure 4C). Live‐cell microplate fluorescence imaging showed a significant decrease in fluorescence intensity in the cells treated with siCDK4, compared with that in cells treated with the control sequence (40.30%, *p *< 0.01) (Figure 4D,E). In addition, laser confocal scanning microscopy also showed a clear reduction in the fluorescence intensity of siCDK4‐treated MCF‐7 cells (Figure 4F,G). These findings showed that CPP30‐Lipo/CDKACT4 is sufficiently sensitive to monitor the transition of the cell cycle from interphase to mitosis and the relative abundance of CDK4 as well as its kinase activity in living cells.

### 2.4 Imaging CDK4 inhibitor pharmacodynamics in living cells

**FIGURE 5:**
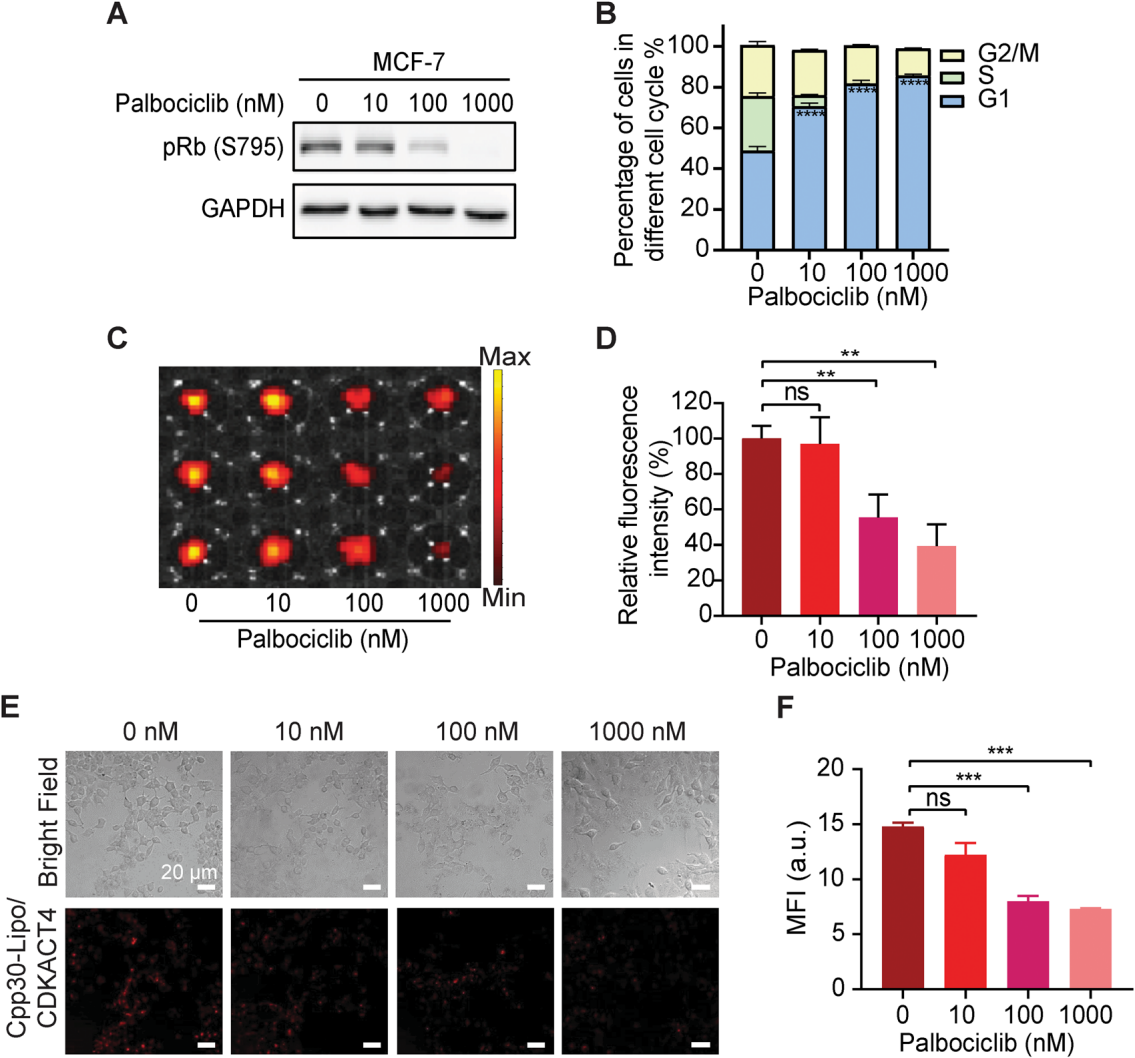
CPP30‐Lipo/CDKACT4 reports the CDK4 inhibitor pharmacodynamics in living cells. (A) Western blot of pRb (S795) protein levels from MCF‐7 cells treated with different concentrations of palbociclib. (B) Distribution of MCF‐7 cells treated with different concentrations of palbociclib in different phases of the cell cycle (*n* = 3). (C, D) Fluorescence images (C) and MFI (D) of MCF‐7 cells treated with different concentrations of palbociclib after incubation with CPP30‐Lipo/CDKACT4 (*n* = 3, **p < 0.01). (E, F) Representative fields under fluorescence microscopy (E) and MFI (F) of MCF‐7 cells treated with different concentrations of palbociclib after incubation with CPP30‐Lipo/CDKACT4 (*n* = 3, ***p < 0.001). Scale bar = 20 μm

We next investigated whether CPP30‐Lipo/CDKACT4 could be used to monitor the inhibition by the CDK4 inhibitor palbociclib in living cells. Palbociclib caused a dose‐dependent decrease in pRb protein level, namely CDK4 kinase activity in MCF‐7 cells (Figure 5A). Apart from that, as the concentration of palbociclib increased, up to 86.39% of cells were arrested in the G1 phase (Figure 5B). When we applied CPP30‐Lipo/CDKACT4 to visualize the inhibition of CDK4 kinase activity by palbociclib, the dose‐dependent reduction of fluorescence intensity, namely, 3.17% for 10 nM, 44.70% for 100 nM (*p* < 0.01), and 60.67% for 1000 nM (*p *= 0.001) (Figure 5C,D), coincided with the decrease of pRb (S795) protein. At the same time, we saw a significant dose‐dependent reduction of fluorescence emission under a microscope (Figure 5E,F). Similarly, in the breast cancer cell line T‐47D, which is also characterized by HR‐positive and HER2‐negative, the fluorescence intensity of CPP30‐Lipo/CDKACT4 decreased with the increasing dose of palbociclib (Figure S3A–C). Generally, the change in fluorescence intensity of CPP30‐Lipo/CDKACT4 was consistent with the change in pRb protein level and cell cycle distribution. This demonstrated that this probe can monitor the CDK4 inhibition of palbociclib in living HR^+^/HER2^–^ cells. Further, we applied CPP30‐Lipo/CDKACT4 probe to visualize the inhibition of CDK4 kinase activity by abemaciclib, which was reported as higher IC50 (2 nM for CDK4) than palbociclib (11 nM for CDK4).^19^ A more obvious dose‐dependent reduction of fluorescence intensity, namely, 14.37% for 10 nM, 50.13% for 100 nM (*p* < 0.01), and 81.35% for 1000 nM (*p* < 0.001) was observed (Figure S3D,E).

### 2.5 Imaging CDK4 inhibitor pharmacodynamics *in vivo*

To image the CDK4 inhibitor pharmacodynamics *in vivo*, the *in vivo* biosafety of CPP30‐Lipo/CDKACT4 was evaluated in healthy BALB/c mice after intravenous injection of the probe. During the 28‐day observation, the mouse body weight, hepatic and renal function, blood cells, and pathology of the main organs did not differ significantly between the control and CPP30‐Lipo/CDKACT4 groups, suggesting good biosafety of CPP30‐Lipo/CDKACT4 (Figure S5).

**FIGURE 6:**
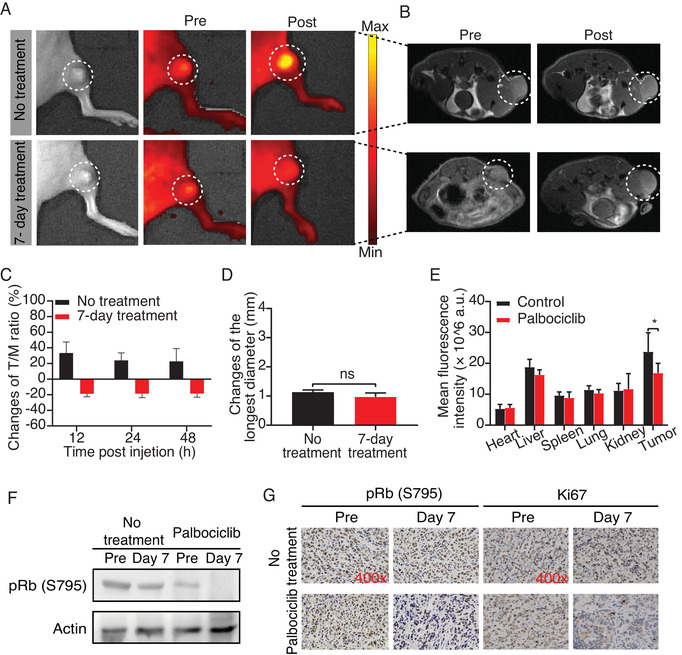
CPP30‐Lipo/CDKACT4 reports the CDK4 inhibitor pharmacodynamics *in vivo*. (A) Fluorescence images of mice bearing MCF‐7 tumor injected with CPP30‐Lipo/CDKACT4 before and after gavage of sterile water for 7 days, or before and after treatment with 150 mg/kg palbociclib daily for 7 days. The fluorescence signals were measured in radiance counts per cm^2^ per second per steradian (p/s/cm^2^/sr). (B) Magnetic resonance imaging (MRI) of mice in (A). (C) Quantitative analysis of the changes of tumor/muscle fluorescence signal ratio between pre and post in the control and palbociclib groups at different time points after caudal vein injection of CPP30‐Lipo/CDKACT4 (*n* = 6). (D) Changes in the longest diameter of tumors treated with water or palbociclib for 7 days (*n* = 6; ns, not significant). (E) The biodistribution of CPP30‐Lipo/CDKACT4 in the heart, liver, spleen, lung, kidney, and tumor from MCF‐7 tumor‐bearing mice with or without 7 days of treatment with palbociclib 48 h post‐injection (*n* = 3, *p < 0.05). (F) Western blot of pRb (S795) protein levels from MCF‐7 tumors treated with palbociclib for 7 days. (G) Immunohistochemical staining of pRb (S795) and Ki67 in MCF‐7 tumors treated with palbociclib for 7 days

Subsequently, we developed MCF‐7 tumor xenografts in BALB/c nude mice and subjected them to daily treatment with 150 mg/kg palbociclib in the experimental group or sterile water in the control group. Then MRI and fluorescence imaging were performed on the 0th and 7th days after daily administration. Fluorescence imaging showed that, after the palbociclib treatment, there was a significant decline of fluorescence intensity in the tumors of the palbociclib group, but a clear increase in the tumors of mice treated with sterile water (Figure 6A). Moreover, the tumor/muscle (T/M) fluorescence signal ratio at different time points after caudal vein injection of CPP30‐Lipo/CDKACT4 decreased significantly in the mice treated with Palbociclib for 7 days (–18.86% on average at 12 h, –18.69% on average at 24 h, and –18.49% on average at 48 h; *n* = 6) (Figure 6C). In contrast, the T/M ratio in mice with no drug treatment presented an increase (33.37% on average at 12 h, 23.94% on average at 24 h, and 22.67% on average at 48 h) (Figure 6C). However, at the same time, when we utilized MRI to evaluate the therapeutic effect of palbociclib by measuring the longest diameter of the tumor, the results showed that there was no significant change in any group (Figure 6B,D). This suggested that CPP30‐Lip/CDKACT4‐based fluorescence imaging can monitor the therapeutic response of palbociclib, which cannot be identified by MRI at the early phase of treatment. In addition, we found no significant difference in tumor volume between the two groups during the 7 days of treatment (Figure S4), suggesting that the change in fluorescence signal was not due to the difference in tumor volume.

Next, the mice were sacrificed and their tumors and main organs were collected for immediate *ex vivo* fluorescence imaging for biodistribution analysis. Results showed that the CPP30‐Lip/CDKACT4 was accumulated more in tumor tissue than elsewhere (Figure 6E), demonstrating its tumor‐targeting ability. More to the point, the fluorescence intensity of tumors treated with palbociclib for 7 days was significantly lower than that of tumors in the control group (Figure 6E). Further, the total tumor homogenates were prepared for western‐blot and immunohistochemical staining of phospho‐Rb (S795) and Ki67. There were clear reductions in the percentage of cells positive for pRb between baseline and day 7 in the palbociclib group, whereas pRb staining was largely preserved in the control group. Similarly, Ki‐67 staining was substantially reduced in the palbociclib group (Figure 6F, G). These results demonstrated that CPP30‐Lip/CDKACT4 can monitor the antiproliferative effect of palbociclib at an early disease stage, accurately, and in real‐time *in vivo*. Besides, although the volume of tumors did not change obviously during the first 7 days of treatment, we observed that tumor growth was significantly inhibited after 14 days of treatment with palbociclib, which was further proof of its effectiveness (Figure S4).

## 3 DISCUSSION

The emergence of targeted drugs raises the need for new strategies to probe relevant target‐specific biomarkers at an early stage and monitor their response to targeted therapeutics in breast cancer. Protein kinases are the most intensively studied category of drug targets in pharmacological research and clinical treatments.^20^ To facilitate the development and clinical implementation of kinase‐targeting treatments, it is essential to have an accurate, non‐invasive, and convenient means of measuring protein kinase activity. In the present study, we developed a novel fluorescent probe, CPP30‐Lipo/CDKACT4, featuring the CDKACT4 peptide biosensor as a response element and the tumor­specific homing and lysosome‐escaping CPP30‐Liposome as a carrier. As expected, this innovative strategy provided a visible readout of CDK4‐CyclinD activity and the inhibition of CDK4 activity by CDK4/6 inhibitors directly and accurately in living cells and *in vivo*.

With regard to the evaluation of CDK4/6 inhibitors, a few clinical studies focusing on molecular imaging have demonstrated methods that can predict the clinical benefits of CDK4/6 inhibition with low or no invasiveness. For instance, Leonard et al. determined the pharmacodynamics of palbociclib using ^18^F‐FDG PET in mantle cell lymphoma.^21^ However, in their study, the metabolic response by FDG‐PET has not correlated with tumor proliferation and CDK4/6 inhibition in some patients whose resistance to palbociclib occurs within several months. This could be explained by the increased activation of CDK2 in the process of palbociclib resistance,^22^, ^23^ and tumor metabolism not completely reflecting the true condition of CDK4/6. Therefore, measuring kinase activity may be the most direct way of evaluating the efficacy of kinase‐targeting agents. Against this background, we developed a new fluorescent probe, which contained an Rb substrate peptide that can be recognized specifically by CDK4‐Cyclin D. We show here that the change in fluorescence intensity of the probe was well consistent with the change in CDK4 activity analyzed by Western blot and immunochemistry. This offers a straightforward and sensitive means of monitoring kinase activity in a continuous fluorescence‐based way.

Bioluminescence imaging has emerged as a sensitive technology to advance our understanding of disease mechanisms at the molecular level and accelerated drug development.^24^, ^25^ We previously reported a p27‐luciferase (p27Luc) fusion protein for monitoring CDK2 activity and the response to CDK2 inhibitory drugs (flavopiridol and roscovitine).^26^ A major shortcoming of this technology is its limitation to perform imaging in the human body because of the need to stably transfect plasmids to cells. The fluorescence imaging based on an exogenous contrast agent developed in the present study may have better biosafety for *in‐vivo* imaging. The good biocompatibility and biosafety of CPP30‐Lipo/CDKACT4 were confirmed both *in vitro* and *in vivo*.

Environment‐sensitive fluorophores are a special class of molecules that exhibit a change in their spectroscopic properties (e.g. fluorescence lifetimes, emission wavelength, and quantum yield) in response to changes in the polarity of their immediate environment.^27^ A wide variety of biosensors have been developed by using environment‐sensitive fluorophores to probe kinase activities, such as PKC,^28^ Akt,^29^ ERK1/2,^30^ and Abl.^31^ Morris et al. described the first fluorescent CDK4/Cyclin D‐specific biosensor by using kinase‐substrate interactions and environment‐sensitive fluorophore 5‐TAMRA.^12^ However, none of them has been applied to measure kinase activity *in vivo*. Rapid blood clearance, poor cellular penetration, and lysosomal degradation of peptide biosensors are major obstacles to *in‐vivo* imaging. Liposomes have attracted substantial attention as pharmaceutical carriers, which can provide increased stability, prolonged circulation times, and protection from degradation.^15^ Among them, cationic liposomes can form ion pairs with anionic lipids within the endosomal membrane, which form the inverted hexagonal (HII) phase and destabilize the endosome membrane, facilitating the endosomal escape of nucleic acid and peptides.^32^, ^33^ DOPE is a neutral phospholipid that has also been confirmed to mediate endosomal escape at acidic pH.^16^, ^34^ In the present study, we took the DOPE‐incorporating cationic liposomes as a carrier for the CDKACT4 biosensor peptide, which enables a successful lysosomal escape to deliver the peptide into cells and maintain its function to monitor the progression of the cell cycle and quantify the change of CDK4 protein level in living cells, especially *in vivo*.

CPPs possess good biosafety and have been included in many clinical trials in the form of CPP‐conjugated therapeutics.^17^ CPP30 exhibits specific targeting of breast cancer cell lines as screened from a peptide library.^18^ Modifying the liposome with CPP30 may enhance the long‐circulating delivery specifically to breast cancer. Our results showed here that liposomes modified with CPP30 could be internalized more into breast cancer cells than the unmodified liposomes, which is consistent with the previous report.

This study is also associated with some limitations. For example, the 5‐TAMRA labeled on CDKACT4 is a fluorophore emitting light in the visible spectra (400–700 nm). In this range, biological compounds and tissues, such as blood, fat, and skin, absorb and scatter incident light to a high degree.^35^ Therefore, CPP30‐Lipo/CDKACT4 is faced with a low signal‐to‐noise ratio and low imaging resolution in *in‐vivo* fluorescent imaging. Further improvements to the probe are thus needed to increase the imaging quality.

In conclusion, we developed a novel peptide biosensor‐based nano‐probe and succeeded in using it to monitor the cancer‐associated CDK4 kinase activity and the therapeutic response to the CDK4/6 inhibitor in living cells and *in vivo*. In view of its performance in providing functional information about kinase activity, CPP30‐Lipo/CDKACT4 may be promising as an alternative approach for analyzing morphology and histology of clinical cases, as well as for drug development.

## 4 MATERIALS AND METHODS

### 4.1 Synthesis of CDKACT4 peptide biosensor

The peptide biosensor CDKACT4 (GFARVYMSRSSGWERPSGGYKF**K**SSPLRIPG) consists of a substrate sequence (GGYKF**K**SSPLRIPG) derived from Rb in which the proline at two residues upstream of the SP phosphorylation site 795 was replaced by a lysine residue and a phospho‐amino‐acid‐binding domain (GFARVYMSRSSGWERPSGG) derived from the interface of Pin1 WW domain with a phosphorylated substrate peptide. Then the hybrid peptides were linked with 5‐TAMRA at the lysine residue. The processes of synthesis and purification were performed in accordance with the previous study^12^ and completed by GL Biochem (Shanghai, China).

### 4.2 Preparation and characterization of CPP30‐Lipo/CDKACT4

Synthesis of DOPE‐PEG_2000_‐CPP30: DOPE‐PEG_2000_‐Maleimide (Avanti Polar Lipids) was mixed with C‐CPP30 (N→C:C‐RLYMRYYSPTTRRYG; GL Biochem) at a 1:1 molar ratio in HEPES buffer (pH  =  6.5). This reaction mixture was gently stirred at 4°C for 48 h under nitrogen gas. The resulting reaction mixture was then placed in a dialysis bag (molecular weight cutoff  =  3500 Da) and dialyzed in deionized water for 48 h to remove the free CPP30. The final solution in the dialysis bag was lyophilized and analyzed by Autoflex maX MALDI‐TOF MS (Bruker).

Liposome preparation: DOPE:DOTAP:cholesterol (10:1:1, mass ratio) was added to a tube and dissolved in chloroform. Then the mixture was dried by a rotary evaporator at 40°C. The dried lipid film was then hydrated with 1 ml of HEPES–dextrose–urea buffer (10 mM HEPES, 5% dextrose, 7 M urea, pH 7.2) containing CDKACT4 peptide and DOPE‐PEG_2000_‐CPP30 (dissolved in dimethyl sulfoxide, DMSO). After bath sonication for 5 min at 40°C, the liposomes were then further downsized by extrusion through 200 and 100 nm polycarbonate membranes, sequentially. Urea, free peptides, and a trace of the DMSO derived from DOPE‐PEG_2000_‐CPP30 stock solution were finally removed by dialysis of liposomes against HEPES–dextrose buffer three times in 24 h using an 8–14 kDa molecular weight cutoff dialysis tube to obtain the product CPP30‐Lipo/CDKACT4. The morphology of CPP30‐Lipo/CDKACT4 was observed by transmission electron microscopy (Hitachi) with an acceleration voltage of 100 kV. The diameter distribution, PDI, and zeta potential of CPP30‐ Lipo/CDKACT4 were obtained through a laser particle sizer (Brookhaven).

### 4.3 Cell lines

The HR^+^/HER2^–^ human breast cancer cell line MCF‐7 was purchased from Deutsche Sammlung von Mikroorganismen und Zellkulturen. The HR^+^/HER2^–^ human breast cancer cell line T‐47D was purchased from American Type Culture Collection. Dulbecco's modified Eagle's medium (DMEM) containing 10% FBS was used for cell culture at 37°C under a 5% CO_2_ atmosphere.

### 4.4 Cell internalization

MCF‐7 cells (∼10^5^ cells per well) were seeded in 12‐well plates and cultured overnight for cell attachment. Subsequently, the cells were treated with CDKACT4 or CPP30‐Lipo/CDKACT4 under the equivalent CDKACT4 concentration (3 μM) for 12 h. At predetermined times (0.5, 1, 3, 6, and 12 h), the cells were collected and suspended in a cold PBS buffer and then immediately analyzed by flow cytometry (Beckman Coulter).

For further analysis of the subcellular localization of CPP30‐Lipo‐CDKACT4, MCF‐7 cells (∼10^5^ cells per well) were seeded on poly‐L‐lysine‐coated coverslips in 12‐well plates and grown in fresh medium containing CPP30‐Lipo/CDKACT4. After 12 h of incubation, the cells were washed three times with PBS, fixed with cold 4% paraformaldehyde for 15 min, permeabilized with 0.1% Triton X‐100 in PBS for 30 min, and blocked with 10% goat serum in PBS for 60 min. Subsequently, the cells were incubated overnight at 4°C with anti‐CDK4 rabbit mAb (1:200 dilution, 12,790; Cell Signaling Technology,) and then with goat anti‐rabbit IgG 488 (1:800 dilution, ab150077; Abcam) at room temperature for 1 h. Finally, the coverslips were washed and mounted onto microscope slides using ProLong Gold antifade mountant with DAPI (Beyotime Biotechnology). The slides were observed with an LSM 880+Airyscan confocal microscope (Carl Zeiss Microscopy).

For endosomal escape, MCF‐7 cells (∼10^5^ cells) were seeded in 35 mm confocal dishes and incubated with CPP30‐Lipo/CDKACT4 at 37°C for 4 h. At the determined time, the culture medium was removed and the cells were washed three times with PBS and incubated with LysoTracker Green DND‐26 (50 nM, 40738ES50; Yeasen Biotech), at 37°C for 30 min and then with Hoechst 33342 (10 μg/ml, C0030; Solarbio) for 10 min. Thereafter, the cells were washed three times with PBS and refilled with a fresh culture medium. Fluorescence images were obtained using a confocal microscope (Carl Zeiss Microscopy).

### 4.5 Phase contrast and time‐lapse imaging

MCF‐7 cells (∼10^5^ cells per well) were seeded in confocal dishes and cultured overnight for cell attachment. Then, CPP30‐Lipo‐CDKACT4 diluted in DMEM containing 20% FBS was overlaid onto the cells. The acquisition of images showing the cell cycle phases and CPP30‐Lipo‐CDKACT4 fluorescence time‐lapse microscopy were performed in an LSM 880+Airyscan confocal microscope (Carl Zeiss Microscopy) every 20 min.

### 4.6 siRNA transfections

siRNAs targeting CDK4 (5′‐GAGATTACTTTGCTGCCTTAA‐3′) and control siRNA (5′‐UUCUCCGAACGUGUCACGUTT‐3′) were purchased from GenePharma (Shanghai, China). siRNA transfection was performed with Lipofectamine™2000 (Invitrogen, Thermo Fisher Scientific), in accordance with the manufacturer's instructions. Cells were analyzed 48 h later.

### 4.7 Western blot

Cells or minced tumor tissues were washed three times with ice‐cold PBS and lysed in RIPA buffer containing protease inhibitor cocktail (Roche Molecular Systems) and phosphatase inhibitor cocktail (Bimake). Tissues were further ground using a glass homogenizer on ice. Protein concentrations were determined with a BCA kit (Solarbio). Subsequent steps were performed as recently described.^36^

### 4.8 Cell cycle analysis

Cells were harvested and fixed in 70% ethanol at 4 °C overnight. Then, the samples were incubated in a solution of 10 mg/ml RNase and 1 mg/ml propidium iodide (Yeasen Biotech) at 37 °C for 30 min in the dark. The DNA content was determined using flow cytometry (Becton, Dickinson & Co.).

### 4.9 Cell microplate fluorescence imaging

Cells (∼8 × 10^3^ cells per well) were seeded in black 96‐well plates and incubated with CPP30‐Lipo/CDKACT4 for 12 h after siRNA transfection or treatment with graded concentrations of palbociclib (HY‐50767; Medchemexpress). Then, the cells were washed three times with PBS and refilled with fresh PBS. Cell fluorescence signals were immediately detected using the IVIS Lumina II imaging system (PerkinElmer).

### 4.10 *In‐vivo* fluorescence and magnetic resonance imaging

For MCF‐7 tumor model establishment, 6–8‐week old female BALB/c nude mice purchased from Xiamen University Laboratory Animal Center were first subcutaneously implanted with 60‐day release 17β‐estradiol pellets (Innovative Research of America) in the right side of the neck. Three days later, 2 × 10^6^ MCF‐7 cells were injected subcutaneously in 1:1 PBS:Matrigel (BioCoat) into the right leg of the nude mice. Tumors were measured by caliper two or three times per week.

When MCF‐7 tumors grew to a volume of 75–100 mm^3^, the mice were randomly divided into two groups (n = 6) and given palbociclib (150 mg/kg diluted in 50 nM sodium D‐lactate) or sterile water daily by oral gavage. Magnetic resonance imaging was performed before (0 days) and 7 days after treatment using a 9.4T BioSpec MicroMRI (Bruker), and fluorescence imaging was also performed using the IVIS Lumina II imaging system (PerkinElmer) at the observation time points after intravenous injection with CPP30‐Lipo/CDKACT4 (10 μl/g body weight, containing 30 μM CDKACT4). After imaging, tumor tissue was collected for IHC staining.

### 4.11 Immunohistochemical staining

Tumors were fixed in 4% paraformaldehyde, embedded in paraffin, and cut into 4‐μm slices. Then, the sections were stained with rabbit monoclonal antibody to Rb (Phospho S795) (1:100 dilution, ab47474; Abcam) or Ki67 (1:500 dilution, 9449; CST). Finally, the sections were observed under an optical microscope.

### 4.12 Statistical analysis

Statistical analysis was performed using GraphPad Prism (Version 8.00; GraphPad Software Inc, CA, USA). Data are presented as mean ± standard deviation. Unpaired two‐tailed Student's *t*‐test or one‐way ANOVA followed by Duncan multiple comparison test was used to analyze the statistical significance of differences. A *p*‐value < 0.05 was considered statistical significance.

## CONFLICT OF INTEREST

The authors declare that they have no conflict of interest.

## ETHICS STATEMENT

All mouse experiments and care were performed in accordance with a procedure approved by the Institutional Animal Care and Use Committee of Xiamen University.

## AUTHOR CONTRIBUTIONS

Conceptualization: Guo‐Jun Zhang; Methodology: Yi‐Yang Gao, Rui‐Qin Yang; Formal analysis and investigation: Yi‐Yang Gao, Rui‐Qin Yang, Kang‐Liang Lou, Yong‐Ying Dang, Yuan‐Yuan Dong, Yue‐Yang He, Wen‐He Huang and Min Chen; Writing‐original draft preparation: Yi‐Yang Gao, Rui‐Qin Yang; Writing‐review and editing: Guo‐Jun Zhang, Yi‐Yang Gao; Funding acquisition: Guo‐Jun Zhang; Resources: Guo‐Jun Zhang; Supervision: Guo‐Jun Zhang. All authors reviewed the report and approved the final version.

## Supporting information

Supporting Information

## Data Availability

The datasets generated during and/or analyzed during the current study are available from the corresponding author on reasonable request.
