# Supplementary material for: In vivo visualization of fluorescence reflecting CDK4 activity in a breast cancer mouse model
Source: MedComm (2020). 2022 Jun 10;3(3):e136. doi: 10.1002/mco2.136 (PMC9187519; doi:10.1002/mco2.136)
Supplement: Supplementary file 1 — Supporting Information [file MCO2-3-e136-s001.docx]

**Supplementary Materials for**

**Title: *In Vivo* Visualization of Fluorescence Reflecting CDK4 Activity in a Breast Cancer Mouse Model**

**Running title: Visualization of CDK4 Activity**

Yi-Yang Gao ^1, 2, 3, 5^, Rui-Qin Yang ^1, 2, 3, 5^, Kang-Liang Lou ^1, 2, 3, 5^, Yong-Ying Dang ^1, 2, 3, 5^, Yuan-Yuan Dong ^1, 2, 3, 5^, Yue-Yang He ^1, 2, 3, 5^, Wen-He Huang ^1, 2, 3, 4^, Min Chen ^3, 5, 6^, Guo-Jun Zhang ^1, 2, 3, 4, 6^

1. Cancer Center & Department of Breast and Thyroid Surgery, Xiang’an Hospital of Xiamen University, School of Medicine, Xiamen University, Xiamen 361101, China
2. Fujian Key Laboratory of Precision Diagnosis and Treatment in Breast Cancer (Xiang'an Hospital of Xiamen University), Xiamen 361101, China
3. Xiamen Key Laboratory of Endocrine-Related Cancer Precision Medicine， Xiang’an Hospital of Xiamen University, Xiamen 361101, China
4. Xiamen Research Center of Clinical Medicine in Breast & Thyroid Cancers, Xiang’an Hospital of Xiamen University, Xiamen 361101, China
5. Central Laboratory, Xiang’an Hospital of Xiamen University, Xiamen 361101, China
6. Cancer Research Center, School of Medicine, Xiamen University, Xiamen 361101, China

Yiyang Gao and Ruiqin Yang contributed equally to this work.

**Correspondence**

Guo-Jun Zhang, MD, PhD, Cancer Research Center and the Department of Breast-Thyroid-Surgery, Xiang’an Hospital of Xiamen University, Xiamen, Fujian, China; Email: [gjzhang@xah.xmu.edu.cn](mailto:gjzhang@xah.xmu.edu.cn), Tel: 0086-592-2889988.

1. **Methods**
   1. **Stability evaluation**

To evaluate the stability of CPP30-Lipo/CDKACT4 in aqueous solution, samples were stored in the dark at room temperature for 96 h. At predetermined times, UV–vis absorption spectra of the solutions were measured by a UV–vis spectrometer (Agilent, Santa Clara, CA, USA) and fluorescence emission spectra were scanned with a fluorescence spectrometer (PerkinElmer, Waltham, MA, USA) at an excitation wavelength of 556 nm.

- 1. **CPP30-Lipo/CDKACT4 biosafety evaluation**

BALB/c mice (4–6 weeks) were randomly divided into two groups (n = 20 each group) and intravenously injected with PBS (200 μL) or CPP30-Lipo/CDKACT4 (30 μM, 10 μL/g). Body weights were measured every day. The mice were sacrificed on days 1, 3, 7, and 28 post-injection. Blood samples and major organs (heart, liver, spleen, lung, kidney and brain) were collected at the selected points. Indexes of hepatic and renal function, including alanine aminotransferase (ALT), aspartate aminotransferase (AST), alkaline phosphatase (ALP), blood urea nitrogen (BUN) and serum creatinine (Scr), as well as blood indexes, including white blood cell (WBC) count, lymphocyte count (Lymph#), red blood cell (RBC) count, and platelet (PLT) count, were analyzed. H&E staining of major organs was performed for histological analysis.

1. **Figures**


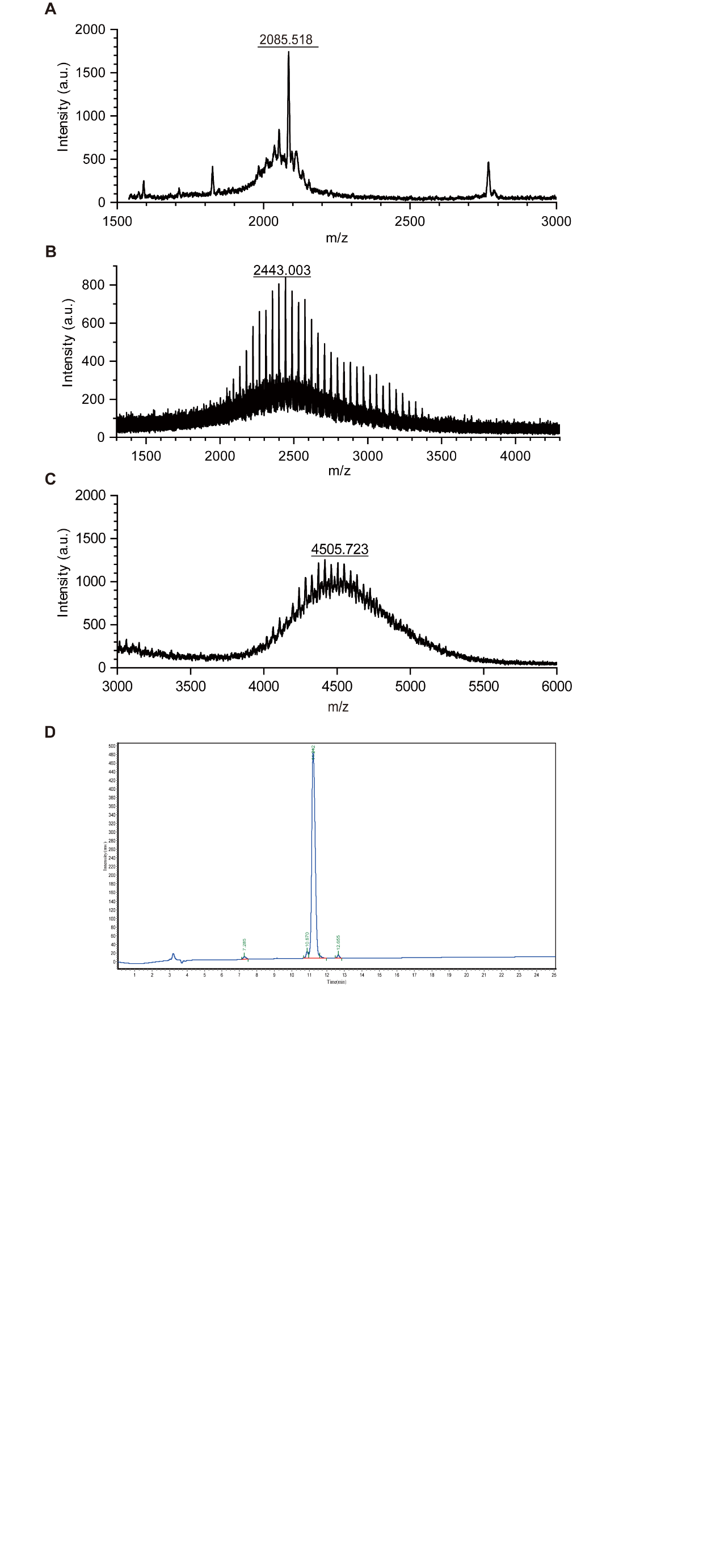


**Figure S1:** The MALDI-TOF-MS of CPP30 (A), DOPE-PEG2000-Maleimide (B) and DOPE-PEG2000-Mal-CPP30 (C). D, HPLC analysis of CDKACT4.


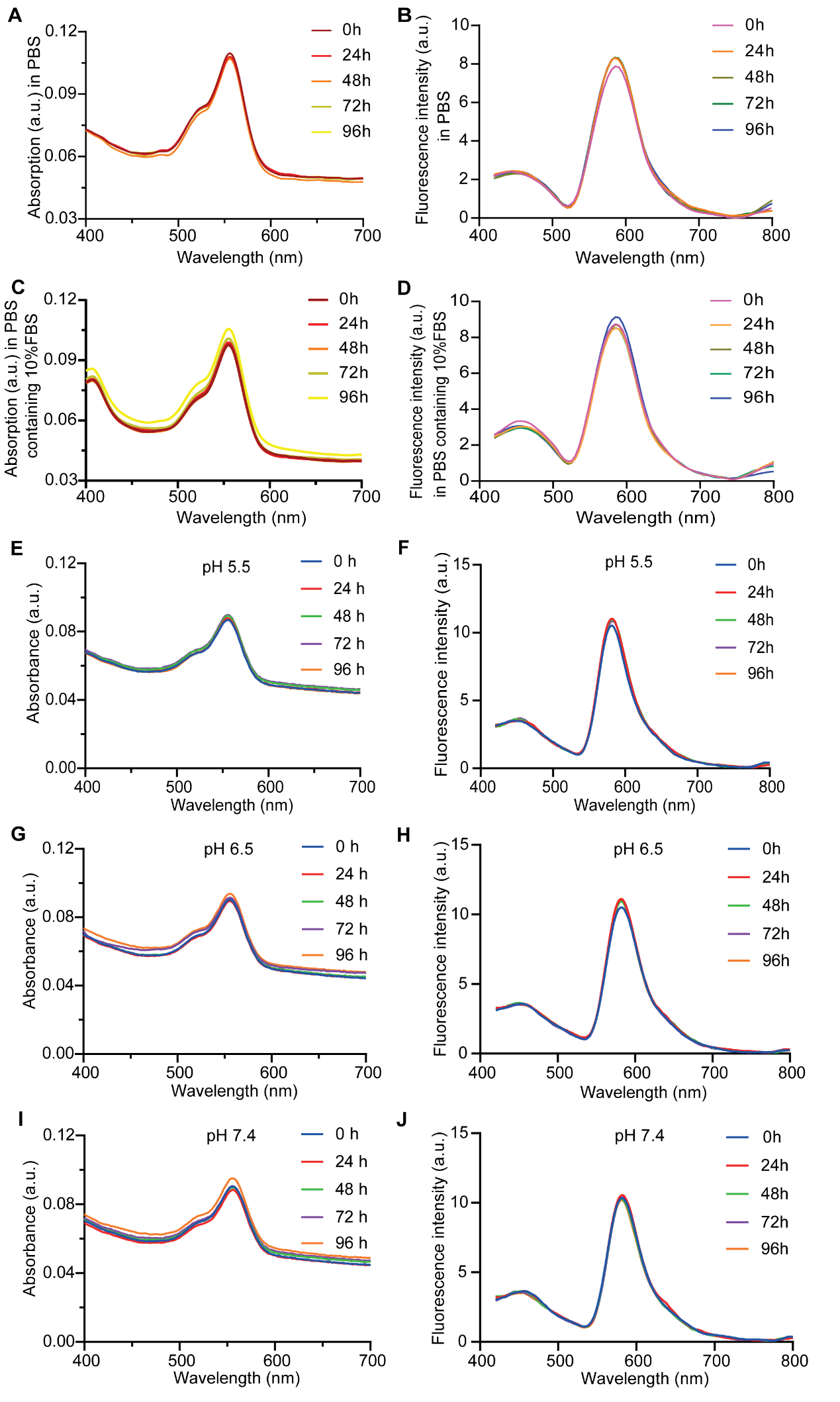


**Figure S2:** Spectral stability of CPP30-Lipo/CDKACT4. A, B, Changes of absorption and fluorescence spectra of CPP30-Lipo/CDKACT4 during 96 h in PBS. C, D, Changes of absorption and fluorescence spectra of CPP30-Lipo/CDKACT4 during 96 h in PBS containing 10%FBS. E, F, Changes of absorption and fluorescence spectra of CPP30-Lipo/CDKACT4 during 96 h at pH 5.5. G, H, Changes of absorption and fluorescence spectra of CPP30-Lipo/CDKACT4 during 96 h at pH 6.5. I, J, Changes of absorption and fluorescence spectra of CPP30-Lipo/CDKACT4 during 96 h at pH 7.4. The samples were stored at room temperature in a dark place.


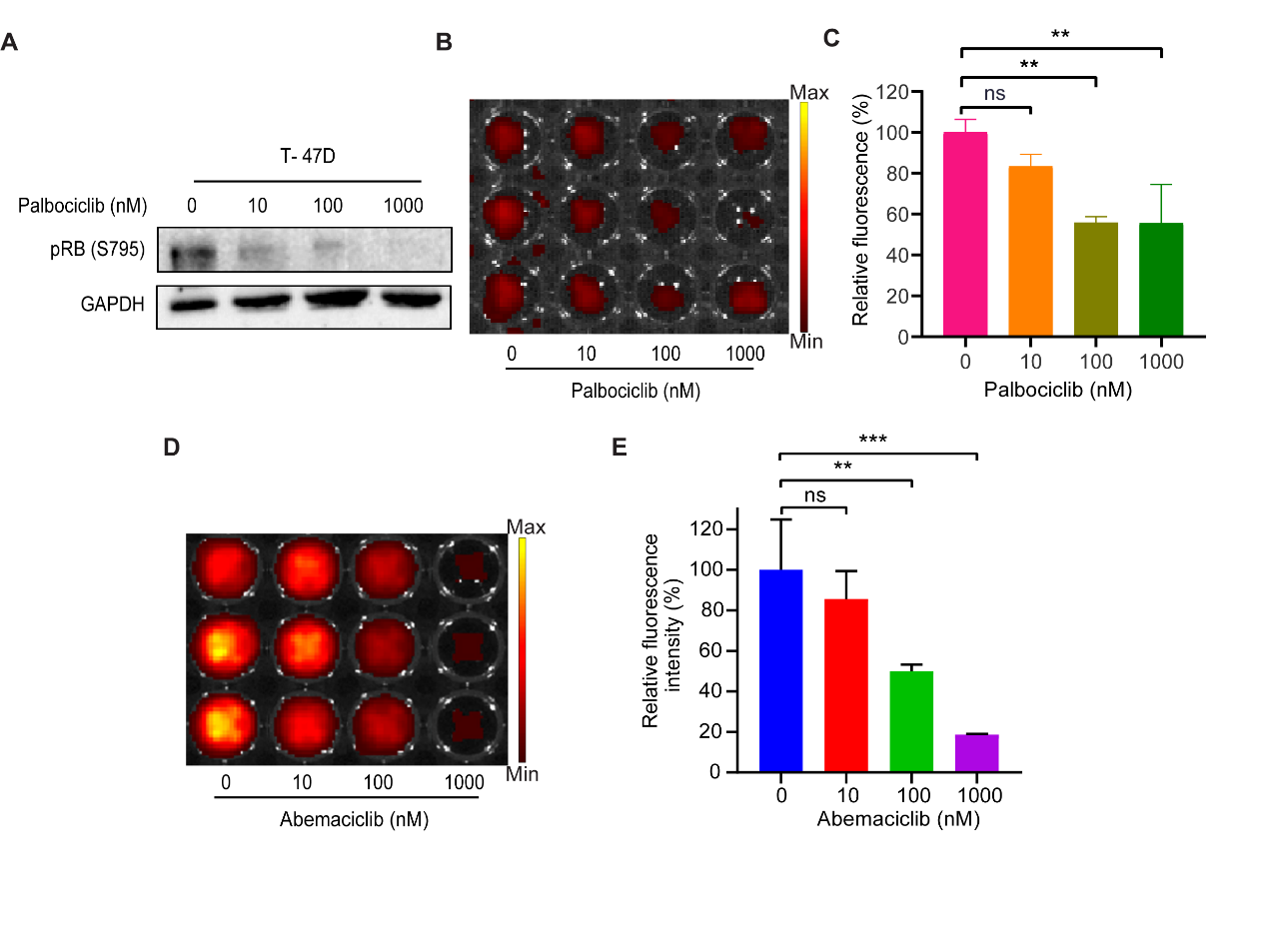


**Figure S3:** CPP30-Lipo/CDKACT4 reports the CDK4/6 inhibitors pharmacodynamics in living cells. A, Western blot of pRb (S795) protein levels from T-47D cells treated with different concentration of palbociclib. B, C, Fluorescence images (B) and MFI (C) of different concentration palbociclib-treated T-47D cells after incubated with CPP30-Lipo/CDKACT4 (n=3, ***P* < 0.01). D, E, Fluorescence images (D) and MFI (E) of different concentration Abemaciclib-treated MCF-7 cells after incubated with CPP30-Lipo/CDKACT4 (n=3, ***P* < 0.01, ****P* < 0.001).


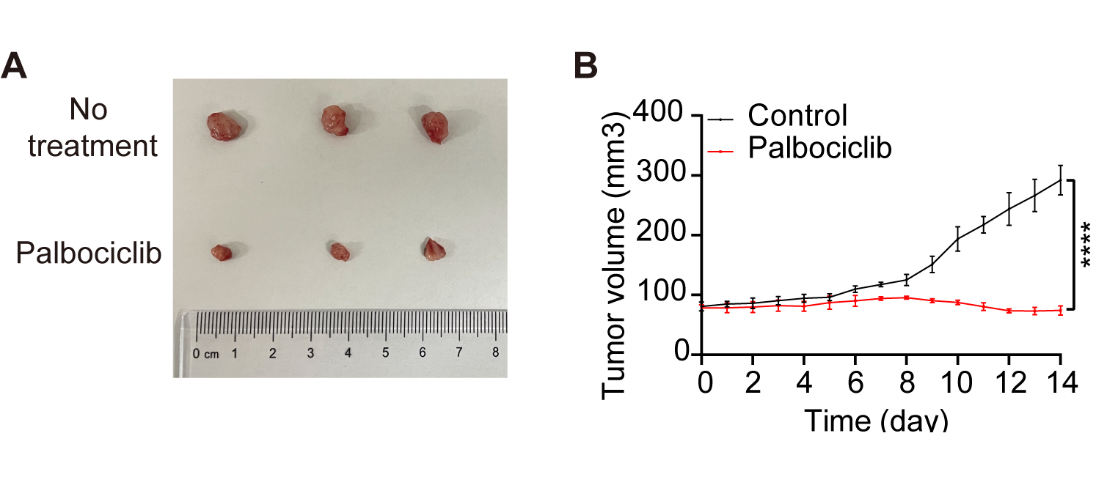


**Figure S4:** *In vivo* antitumor evaluation of palbociclib. A, Tumors of MCF-7 tumor-bearing mice with no treatment or daily palbociclib treatment after 14 days (n = 4). B, Tumor growth curves of MCF-7 tumor-bearing mice within 14 days of treatment (n = 4, *****P* < 0.0001).


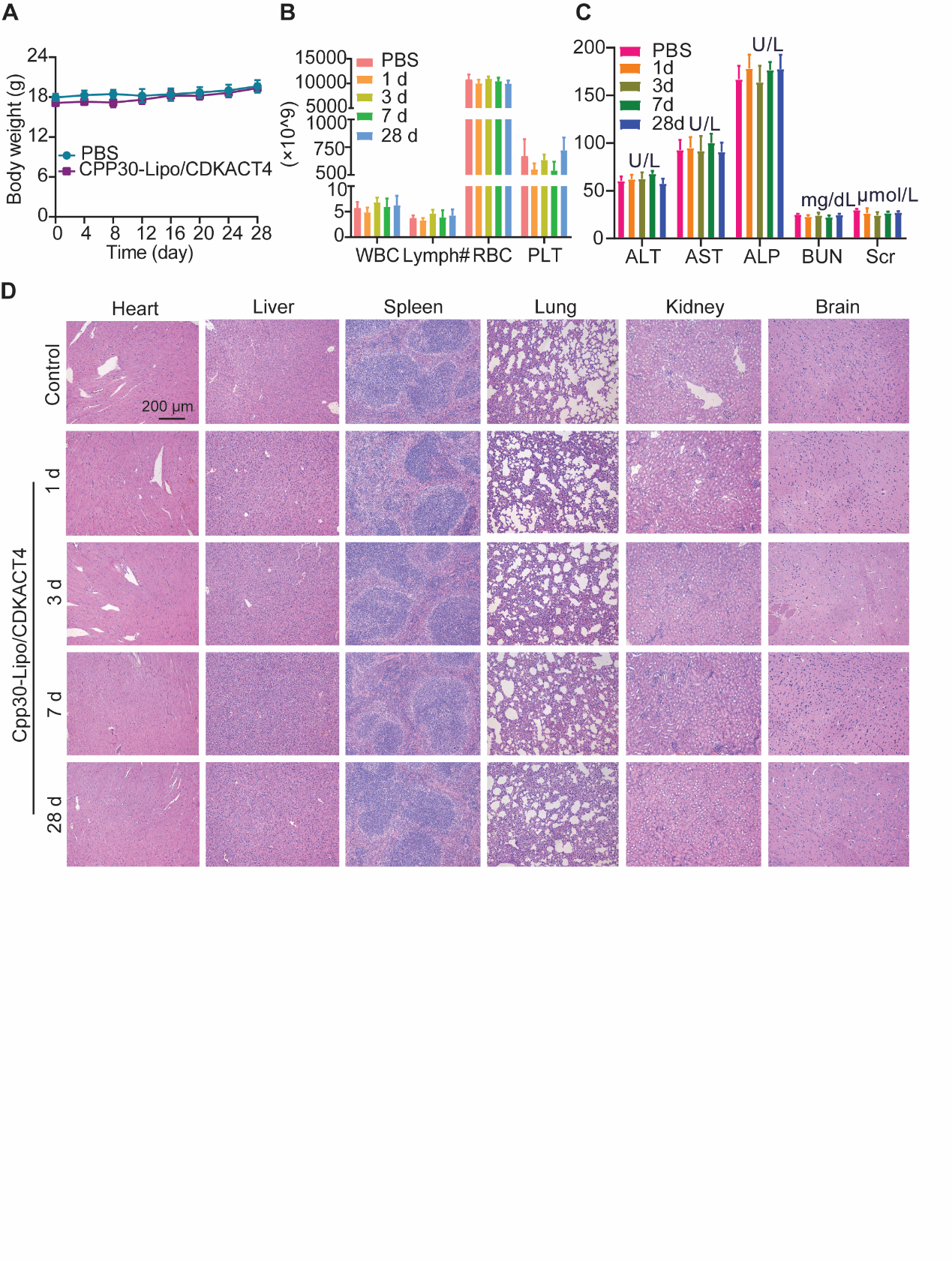


**Figure S5:** *In vivo* biosafety evaluation of CPP30-Lipo/CDKACT4. A, Body weight curves of BALB/c mice within 28 days after caudal vein injection of PBS or CPP30-Lipo/CDKACT4 (n = 5). B, Blood indexes of BALB/c mice after caudal vein injection of PBS or CPP30-Lipo/CDKACT4 for different duration (n = 5). C, Indexes of hepatic and renal functions of BALB/c mice after caudal vein injection of PBS or CPP30-Lipo/CDKACT4 for different duration (n = 5). D, Representative H&E-stained images of organs after caudal vein injection of CPP30-Lipo/CDKACT4 as indicated.

**Table S1:** The Polymer dispersity index (PDI) and Zeta potential of CPP30-Lipo/CDKACT4 particles (n=3)

|  | **PDI** | **Zeta potential（mV）** |
| --- | --- | --- |
| CPP30-Lipo/CDKACT4 | 0.12 | +60.4 |
|  | 0.02 | +60.3 |
|  | 0.08 | +59.9 |
